# Simplified Clinical Decision Rule Using Clinically Important Events for Risk Prediction in Pediatric Head Injury: A Retrospective Cohort Study

**DOI:** 10.3390/jcm10225248

**Published:** 2021-11-11

**Authors:** Naoki Yogo, Chiaki Toida, Takashi Muguruma, Masayasu Gakumazawa, Mafumi Shinohara, Ichiro Takeuchi

**Affiliations:** 1 Department of Emergency Medicine, Graduate School of Medicine, Yokohama City University, 4-57 Urafunecho, Minami-ku, Yokohama 232-0024, Japan; naoki.y0715@gmail.com (N.Y.); mgrmtks@gmail.com (T.M.); msys.gkmzw@gmail.com (M.G.); shinoharamafumi@yahoo.co.jp (M.S.); itake@myad.jp (I.T.); 2 Department of Pediatrics, Division of Pediatric Emergency and Critical Care, Japanese Red Cross Kumamoto Hospital, 2-1-1 Ngamineminami, Higashi-ku, Kumamoto 861-8520, Japan; 3 Emergency Center, Institute of Critical Care, Yokosuka Kyosai Hospital, 1-16 Yonegahamadori, Yokosuka 238-8558, Japan

**Keywords:** pediatrics, emergency department, clinically important traumatic brain injury

## Abstract

Computed tomography (CT) scans are useful for confirming head injury diagnoses. However, there is no standard clinical decision rule (CDR) for determining the need for CT scanning in pediatric patients with head injuries. We developed a CDR and conducted a retrospective cohort study to evaluate its diagnostic accuracy in identifying children with clinically important traumatic brain injury (ciTBI). We selected predictors based on three existing CDRs: CATCH, CHALICE, and PECARN. Of the 2569 eligible patients, 645 (439 (68%) boys, median age: five years) were included in this study. In total, 59 (9%) patients showed ciTBI, and 129 (20%) were admitted to hospital. The novel CDR comprised six predictors of abnormal CT findings. It had a sensitivity of 79.5% (95% confidence interval (CI): 65.5–89.0%) and a specificity of 50.9% (95% CI: 48.9–52.3%). The area under the receiver-operating characteristic curve (0.72, 95% CI: 0.67–0.77) was non-inferior to those of CATCH, CHALICE, and PECARN (0.71, 95% CI: 0.66–0.77; 0.67, 95% CI: 0.61–0.74; and 0.69, 95% CI: 0.64–0.73, respectively; *p* = 0.57). The novel CDR was statistically noninferior in diagnostic accuracy compared to the three existing CDRs. Further development and validation studies are needed before clinical application.

## 1. Introduction

Pediatric head injuries, which are mostly of mild severity, are a frequent cause of visits to the emergency department (ED). However, head injury is a major cause of death in children [1]. Computed tomography (CT) is useful for the confirmation of a diagnosis of head injury, and CT findings constitute the standard diagnostic modality. However, radiation exposure from CT scans might result in lethal malignancies [2,3]; it is thus important to consider the need for CT scans in pediatric patients with head injuries [4,5,6,7,8,9,10,11]. There are three clinical decision rules (CDRs) that have been formulated to prevent a missed diagnosis of serious or clinically important traumatic brain injuries (ciTBI) and to avoid unnecessary CT scans: the Canadian Assessment of Tomography for Childhood Head Injury (CATCH), the Children’s Head Injury Algorithm for the Prediction of Important Clinical Events (CHALICE), and the Pediatric Emergency Care Applied Research Network (PECARN) [4,5,6]. CATCH predicts the need for a CT scan and stratifies a high risk for neurological intervention and a medium risk for possible brain injury on CT. CHALICE is a rule that predicts a low risk of intracranial pathology if none of the variables are present. PECARN defines patients with ciTBI as those in need of neurosurgery, tracheal intubation for >24 h, hospitalization duration >2 days for persistent neurological symptoms or signs, or a fatal outcome; and identifies children who are at a very low risk of ciTBIs. The aim of these rules is to reduce the number of unnecessary CT scans by classifying the risk of a potentially serious head injury. Nonetheless, these rules are cumbersome to use in clinical practice because they include many predictors, and the rules vary according to the patient’s age. In addition, the CATCH and PECARN rules have many inclusion and exclusion criteria. There is no standardized, simple, and accurate assessment procedure that is suitable for determining the need for CT imaging in patients with head injuries across a wide range of pediatric age groups. 

This study was conducted with the aim to develop and validate prediction rules that would be easy to use in clinical practice, which include a small number of predictors and no variations in the rule by age groups. Furthermore, we compared the accuracy of our simplified CDR for identifying pediatric patients with ciTBI with that of the currently available rules such as CATCH, CHALICE, and PECARN. The objective of the study was to create a simple rule that was as accurate as the existing criteria for identifying ciTBI in pediatric head injury patients presenting to the emergency department.

## 2. Materials and Methods

### 2.1. Study Setting and Population

We conducted a retrospective cohort study involving patients <16 years of age with head trauma, who were admitted to five EDs in Japan. The participating hospitals were district general hospitals that each treated between 13,000 and 60,000 emergency patients per year. No established CDR is mandated at any of these hospitals for the assessment of patients with head trauma; the decision on whether to perform a CT scan is made by each physician based on the patient’s clinical characteristics and history.

**Figure 1:**
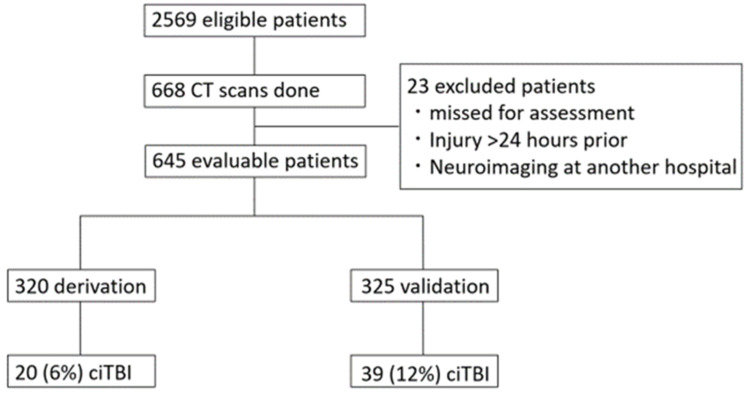
Flow diagram of patient selection. ciTBI, clinically important traumatic brain injury; CT, computed tomography.

Inclusion criteria for this study were: (1) age <16 years; (2) a history of blunt head injury within 24 h before admission to the ED; and (3) undergoing a head CT scan for the first time in the ED. Patients transferred from another hospital after undergoing neuroimaging and those who refused consent for treatment were excluded from this study. Accordingly, we enrolled the derivation population from April 2014 to December 2015 and the validation population from January 2016 to March 2018 (Figure 1).

The primary outcome measure was the diagnostic accuracy (sensitivity, specificity, negative predictive value (NPV), and positive predictive value (PPV) ) of the CDR. Regarding the clinical context, the primary outcome measures in this study were the following PECARN outcomes of ciTBI: those in need of neurosurgery, tracheal intubation for >24 h, hospitalization duration >2 days for persistent neurological symptoms or signs, or a fatal outcome.

### 2.2. Data Collection

Data for demographic variables (age and sex), clinical characteristics (trauma mechanism, clinical history prior to the hospitalization, signs, and management of the patient), and outcome information (ciTBI) were collected from each patient’s electronic medical record. For predictive variables in this study, patients with altered mental status were defined as those with agitation, somnolence, or/and repetitive questioning during verbal communication.

The person in charge of each hospital registered the data with the data center, and then three authors (N.Y., M.G. and M.S.) independently reviewed all the data, selected the study participants, and established the study dataset.

### 2.3. Method of Predictor Selection

We selected potential predictor variables based on the assessment tools such as CATCH, CHALICE, and PECARN that were available in the existing literature. Variables of CATCH, CHALICE, and PECARN were summarized into 17 variables that were specific to this study, and these variables are shown in Appendix A. The original inclusion and exclusion criteria for each rule are shown in Table 1.

### 2.4. Statistical Analysis

Statistical analyses were performed using Stata SE software, Version 16.0 (StataCorp, College Station, TX, USA). Results were expressed as the median and interquartile range for continuous variables, or as frequencies and percentages for categorical variables. The Mann–Whitney U test was used for the analysis of continuous variables, whereas Fisher’s exact test was employed for categorical variables. For all statistical tests, a two-sided *p*-value < 0.05 indicated statistical significance.

We developed a CDR in this study, wherein predictors were selected from potential predictor variables based on the existing literature; if the variables were determined to be statistically significant in the univariate logistic regression analysis, they were included in the multivariable analysis. The accuracies of the rules to predict ciTBI for pediatric patients with head injury were evaluated by calculating the area under the receiver-operating characteristic curve (ROC). Area under the curve (AUC) values for the four rules including the simplified CDR that we developed in this study, CATCH, CHALICE, and PECARN were then compared. Moreover, this study compared the sensitivity, specificity, PPV, and NPV of the four CDRs.

### 2.5. Ethics Statement

The study was approved by the Independent Ethics Committees of the Yokohama City University Medical Center, Yokohama Municipal Citizen’s Hospital, National Hospital Organization Yokohama Medical Center, Yokosuka Kyosai Hospital, and Japanese Red Cross Kumamoto Hospital to maintain patient confidentiality. The requirement of informed patient consent was waived due to the retrospective nature of the study. 

## 3. Results

Of a total of 2569 eligible patients, we included 645 patients in this study, of whom 582 had normal imaging results on CT scanning and 59 had ciTBI (Figure 1).

The patient characteristics are shown in Table 2. Overall, 439 (68%) patients were male; the median age was five years; 129 (20%) children were hospitalized, and nine (1.4%) underwent neurosurgery. The most common injury mechanisms were falls (*n* = 98, 15%), motor vehicle accidents (*n* = 60, 9%), and bicycle accidents while riding without a helmet (*n* = 39, 6%). The history included suspicion of non-accidental injury (*n* = 8, 1%), headache (*n* = 41, 6%), vomiting (*n* = 146, 23%), seizures (*n* = 22, 3%), loss of consciousness (LOC) (*n* = 24, 4%), and amnesia (*n* = 41, 6%).

Findings on examination included a Glasgow Coma Scale (GCS) score <15 (*n* = 72, 11%), irritability (*n* = 2, 0.3%), signs of skull fracture (*n* = 4, 0.6%), hematoma (*n* = 18, 3%), altered mental status (*n* = 127, 20%), and positive focal neurological signs (*n* = 7, 1%).

Univariate analysis was performed using ciTBI as the outcome measure. The six predictors of our CDR comprised: (1) fall from >0.9 m; (2) severe or worsening headache; (3) seizure; (4) LOC; (5) GCS score <15; and (6) altered mental status.

**Figure 2:**
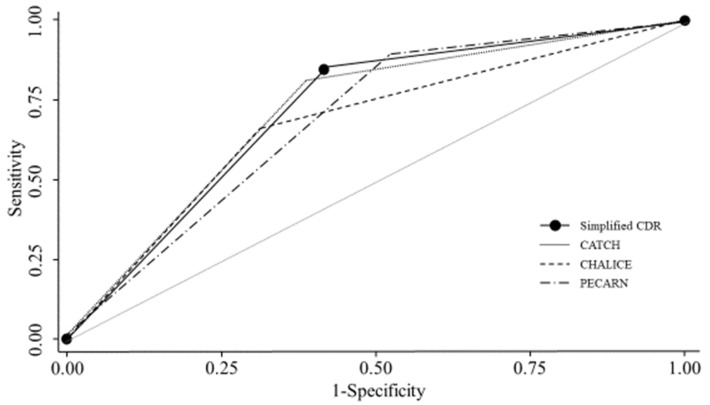
Comparison of our simplified clinical decision rule with CATCH, CHALICE, and PECARN. The novel simplified CDR (AUC 0.72; 95% CI: 0.67–0.77), CATCH (AUC 0.71; 95% CI: 0.66–0.77), CHALICE (AUC 0.67; 95% CI: 0.61–0.74), and PECARN (AUC 0.69; 95% CI: 0.64–0.73) for clinically important TBI. Abbreviations: CATCH, Canadian Assessment of Tomography for Childhood Head Injury; CDR, clinical decision rule; CHALICE, Children’s Head Injury Algorithm for the Prediction of Important Clinical Events; PECARN, Pediatric Emergency Care Applied Research Network; TBI, traumatic brain injury.

When assessing participants who had undergone CT, our simplified CDR in this study had a sensitivity for ciTBI of 79.5% (95% confidence interval [CI]: 65.5–89.0%), a specificity of 50.9% (95% CI: 48.9–52.3%), NPV of 94.4% (95% CI: 90.6–97.0%), and PPV of 19.1% (95% CI: 15.8–21.4%). Table 3 and Figure 2 compare the performance of the novel CDR with CATCH, CHALICE, and PECARN. The ranked sensitivities are as follows: PECARN 89.7% (95% CI: 77.3–95.9%), CATCH 84.6% (95% CI: 71.2–92.6%), novel CDR 79.5% (95% CI: 65.5–89.0%), and CHALICE 64.1% (95% CI: 49.5–76.7%). Ranked specificities were as follows: CATCH 61.0% (95% CI: 59.1–62.2%), CHALICE 60.3% (95% CI: 58.2–62.1%), novel CDR 50.9% (95% CI: 48.9–52.3), and PECARN 39.5% (95% CI: 37.6–40.4%). The AUC for our rule (0.72, 95% CI: 0.67–0.77) was not inferior to those of CATCH, CHALICE, and PECARN (0.71, 95% CI: 0.66–0.77; 0.67, 95% CI: 0.61–0.74; and 0.69, 95% CI: 0.64–0.73, respectively; *p* = 0.57). 

The novel simplified CDR misclassified eight patients with ciTBI as having a low risk (Table 4). The patient who required neurosurgery had an acute epidural hematoma that occurred after the initial CT. Of these eight patients, five cases met the criteria for CATCH, three cases for CHALICE, and six cases for PECARN. The breakdown of these criteria which were met for CATCH, CHALICE, and/or PECARN were: MVC in five cases, fall from bicycle with no helmet in one case, history of vomiting in three cases, and hematoma in one case.

Table 5 shows the ratio of patients with or without ciTBI according to the number of predictors applied by our CDR. As the number of applicable predictors of the CDR increased, the ratio of patients with ciTBI increased and the ratio of patients without ciTBI similarly decreased.

## 4. Discussion

In this retrospective study, we developed and evaluated a CDR consisting of six predictors to detect ciTBI in pediatric patients with head injury. The novel CDR was not statistically inferior in the diagnostic accuracy compared to the three existing CDRs. Moreover, the novel rule has advantages compared to the other three CDRs. First, unlike the CHALICE and PECARN rules [4,5], physicians might not have to change the predictors according to age. Furthermore, our rule has fewer exclusion criteria, which allows the inclusion of almost all children with head injury. Second, the novel CDR is easier to apply in patients with head injury because it comprises fewer predictors and includes only one predictor for the mechanism of injury. 

The three CDRs, CATCH, CHALICE, and PECARN, were derived in large multicenter studies with a high methodological quality. However, they targeted different age groups, and it was not possible to directly compare the three rules. The ages of the targets were less than 17, 16, and 18 years for CATCH, CHALICE, and PECARN, respectively [4,5,6]. In addition, some of the predictors for CHALICE were different for children under one year. Specifically, there are differences in the rule such as different values for GCS, and an additional variable for wound. The rule for PECARN is different for children under two years of age. Given the fact that the predictor varies with age, it is expected to be difficult to use in clinical practice. In this respect, our rule is easier to employ because there are no differences in variables based on age. As exclusion criteria, CHALICE excludes head injury due to abuse, and PECARN excludes a trivial mechanism of injury (ground-level fall, walking, or running into stationary objects, and no signs of head trauma other than scalp abrasions and lacerations). A prospective observational study with 20,137 patients evaluable for analysis showed that there were many patients for whom CATCH and PECARN did not apply. When applying rule-specific inclusion and exclusion criteria, CATCH was applicable for 4957 (25%) patients, and PECARN was applicable for 4011 (75%) patients younger than two years and 11,152 (76%) patients aged two years and older [12]. The absence of such exclusion criteria might make the novel CDR easier to apply in clinical practice.

The criteria of our novel CDR, which consists of only six factors, are more simplified than that of the other three rules. 

CATCH has seven major predictors and ten predictors (four variables of mechanism, one variable of history, and five variables of examination if detailed predictors of mechanism are included); CHALICE has 14 predictors (three variables of mechanism, six variables of history, and five variables of examination); and PECARN has ten predictors (four variables of mechanism, two variables of history, and four variables of examination for children younger than two years; four variables of mechanism, three variables of history, and three variables of examination for two years old or older). Thus, compared to other rules, the novel CDR has fewer predictor variables: one variable related to mechanism, three variables related to history, and two variables related to the findings on clinical examination. Due to the simplified criteria of the novel CDR, our rule can be easily and quickly used for pediatric patients of all age groups in busy emergency room settings. 

A distinguishing feature of our rule is that it includes only one predictor of the mechanism of injury. Pediatric injury often occurs at home. According to the Consumer Affairs Agency of Japan, approximately 60% of children’s accidents occur in homes and roads; similar trends can be observed in developed countries [13]. A previous study reported that the injury mechanism is often unknown because pediatric head injuries often occur in the patient’s home [14]. As a result, there are many cases where there is no witness or only family members respond at the time of injury; therefore, the mechanism of injury is not considered reliable. The actual mechanism of injury is not evident while obtaining the medical history in cases of abusive head trauma. In fact, PECARN rules were also reported to be less sensitive for physically abused children [11]. From these facts, it may be an overestimation to emphasize the injury mechanism as a predictor. 

Furthermore, clinical physician judgement was reported to be equal to or greater than the accuracy of the three CDRs [7,15]. These results emphasize the importance of focusing on physical examinations rather than the mechanism of injury itself. The three CDRs state that CT should not be performed for diagnostic imaging of patients with brain injury who do not need acute intervention. However, methods to minimize unnecessary CT scanning are not specified. In fact, PECARN, which is frequently used with a high accuracy, has the highest frequency of CT scanning among the three CDRs [12]. In addition, strict adherence to the three CDRs may increase the frequency of CT scans; therefore, a CDR that emphasizes physical examination by a physician, as in the novel CDR, is needed. Actually, one patient with ciTBI, who our CDR misclassified as low risk and who later underwent surgical intervention, did not adopt the criteria of our CDR at the time of emergency room consultation. After returning home, this patient underwent CT and a surgical intervention because the vomiting episodes became repeated over time. Thus, a risk classification necessitating follow-up might be required. 

Moreover, because our CDR misclassified eight patients with ciTBI as low risk in this study, the novel CDR might need further improvement. This study showed that the ratio of patients with ciTBI increased according to the number of predictors applied by our CDR (Table 5). This result suggests that our CDR might be able to quantify the degree of risk for patients with ciTBI by quantification index in the next step. 

Our study has several limitations. A direct comparison of rules is not possible because they address different questions (patients who should or should not undergo CT), age groups, injury severities, and outcomes. Therefore, there may be an insufficient matching of the patient background. As this was a small-scale study, it is inadvisable to generalize its outcome to other patient populations. Prospective studies with a large number of participants at different hospitals are needed to validate the findings of this study. Patients who underwent CT were included to avoid missing cases of ciTBI, which may have led to selection bias. Due to the retrospective nature of the study design, we could not meaningfully evaluate the reliability and validity of the data. It is possible that the numerical values of the predictors are not accurately tabulated. Although the decision to perform CT was not made, the widespread use of the three CDRs might influence the physician’s decision and result in bias. These factors may have caused a dissociation between the results as reported in the original papers and this study. In addition, the number of patients in this study was small, and verification by age group was insufficient. This CDR may be useful with ease in clinical practice. However, it may be difficult to judge CT scan by this rule because of its low predictivity. To overcome these limitations, an external validation study will be needed with more sample sizes to achieve a more generalized examination of the accuracy of our CDR. In addition, Japan has the world’s highest rate of CT installation, making it an environment where CT can be easily performed. This makes it easy for even minor abnormal findings to be misdiagnosed. A research plan that takes these factors into account is necessary.

## 5. Conclusions

We detected and validated predictors of ciTBI in children. The novel simplified CDR was compared with CATCH, CHALICE, and PECARN; the accuracy of the novel CDR was non-inferior for identifying pediatric patients with ciTBI. Further development and validation studies are needed before the application of the novel CDR in the clinical setting.

## Figures and Tables

**Table 1:** Comparison of patient selection criteria in the clinical decision rules.

	CATCH	CHALICE	PECARN	Simplified CDR
Inclusion criteria	Age < 17 years	Age < 16 years	Age < 18 years	Age < 16 years
	All the following:	Any history or signs of injury to the head	Presenting within 24 h of head injury	Any of the following:
	▪ Blunt trauma to the head resulting in witnessed LOC/disorientation, definite amnesia, persistent vomiting (≥2 distinct episodes of vomiting 15 min apart), persistent irritability in the ED (in children <2 y)			▪ History of blunt head injury within 24 h before admission to the ED
	▪ Initial GCS in ED >13, as determined by the treating physician			▪ Undergoing CT scanning for the first time in the ED
	▪ Injury within the past 24 h			
Exclusion criteria	Any of the following:	Refusal to consent	▪ Trivial mechanism (defined as ground-level fall, walking or running into a stationary object, no signs or symptoms of head trauma except scalp abrasions and lacerations)	Refusal to consent
	▪ Obvious penetrating skull injury ▪ Obvious depressed fracture ▪ Acute focal neurological deficit ▪ Chronic generalized developmental delay		▪ Penetrating trauma	
	▪ Head injury secondary to suspected child abuse		▪ Known to have a brain tumor	
	▪ Returning for reassessment of previously treated head injury		▪ Preexisting neurological disorder complicating assessment	
	▪ Patients who were pregnant		▪ Neuroimaging at another hospital before transfer	
			▪ Patient with ventricular shunt	
			▪ Patient with bleeding disorder	
			▪ GCS < 14	

Abbreviations: CATCH, Canadian Assessment of Tomography for Childhood Head Injury; CDR; clinically decision rule; CHALICE, Children’s Head Injury Algorithm for the Prediction of Important Clinical Events; ED, emergency department; GCS, Glasgow Coma Scale; LOC, loss of consciousness; PECARN, Pediatric Emergency Care Applied Research Network.

**Table 2:** Distribution of demographic variables, predictive variables, and outcomes.

	All Patients (*n* = 645)	Derivation (*n* = 320)	Validation (*n* = 325)	*p*-Value
Demographic variables				
Age in years, median (IQR)	5 (2–9)	5 (2–8)	6 (2–10)	<0.05
Male, *n* (%)	439 (68)	217 (68)	222 (68)	0.93
Predictor variables, *n* (%)				
Mechanism				
Motor vehicle accident	60 (9)	19 (6)	41 (13)	<0.05
Bicycle accident without helmet	39 (6)	21 (7)	18 (6)	0.87
Fall from >0.9 m	98 (15)	51 (16)	47 (15)	1
Fall from ≥5 stairs	40 (6)	25 (8)	15 (5)	0.19
High-impact striking of the head against an object	5 (0.8)	0 (0)	5 (2)	<0.05
History				
Suspicion of non-accidental injury	8 (1)	1 (0.3)	7 (2)	<0.05
Severe or worsening headache	41 (6)	18 (6)	23 (7)	0.42
Vomiting	146 (23)	67 (21)	79 (24)	0.16
Seizure	22 (3)	12 (4)	10 (3)	0.83
Amnesia	41 (6)	12 (4)	29 (9)	<0.05
Loss of consciousness	24 (4)	9 (3)	15 (5)	0.21
Examination				
Glasgow Coma Scale score <15	72 (11)	33 (10)	39 (12)	0.26
Irritability on examination	2 (0.3)	1 (0.3)	0 (0)	1
Any signs of skull fracture	4 (0.6)	4 (1)	0 (0)	0.12
Hematoma	18 (3)	10 (3)	8 (3)	0.81
Altered mental status	127 (20)	42 (13)	85 (26)	<0.05
Positive focal neurology	7 (1)	1 (0.3)	6 (2)	0.06
Outcomes, *n* (%)				
Abnormality on CT scan	63 (10)	26 (8)	37 (11)	0.19
Neurosurgery	9 (1)	3 (0.9)	6 (2)	0.33
Hospital admission	129 (20)	50 (16)	79 (24)	<0.05
Death secondary to head injury	0 (0)	0 (0)	0 (0)	1
ciTBI	59 (9)	20 (6)	39 (12)	<0.05

Abbreviations: CT, computed tomography; IQR, interquartile range; ciTBI, clinically important traumatic brain injury.

**Table 3:** Performance of the clinical decision rules for clinically important traumatic brain injury.

	Sensitivity (%)	Specificity (%)	NPV (%)	PPV (%)	AUC
	(95% CI)	(95% CI)	(95% CI)	(95% CI)	(95% CI)
Simplified CDR	79.5 (31/39)	50.9 (136/267)	94.4 (136/144)	19.1 (31/162)	0.72
	(65.5–89.0)	(48.9–52.3)	(90.6–97.0)	(15.8–21.4)	(0.67–0.77)
CATCH	84.6 (33/39)	61.0 (163/267)	96.4 (163/169)	24.1 (33/137)	0.71
	(71.2–92.6)	(59.1–62.2)	(93.4–98.3)	(20.3–26.4)	(0.66–0.77)
CHALICE	64.1 (25/39)	60.3 (161/267)	92.0 (161/175)	19.1 (25/131)	0.67
	(49.5–76.7)	(58.2–62.1)	(88.7–94.8)	(14.7–22.8)	(0.61–0.74)
PECARN	89.7 (35/39)	39.7 (106/267)	96.3 (106/110)	17.9 (35/196)	0.69
	(77.3–95.9)	(37.6–40.4)	(91.9–98.5)	(15.4–19.1)	(0.64–0.73)

Abbreviations: AUC, area under the receiver-operating characteristic curve; CATCH, Canadian Assessment of Tomography for Childhood Head Injury; CDR, clinical decision rule; CI, confidence interval; CHALICE, Children’s Head Injury Algorithm for the Prediction of Important Clinical Events; NPV, negative predictive value; PECARN, Pediatric Emergency Care Applied Research Network; PPV, positive predictive value.

**Table 4:** Demographic characteristics of patients with ciTBI without simplified CDR predictors.

Age (Years)	Sex	Final Diagnosis	Treatment	Meeting the Criteria of CATCH	Meet the Criteria of CHALICE	Meet the Criteria of PECARN	Breakdown of the Criteria for CATCH, CHALICE, and/or PECARN			
							MVC	Fall from a Bicycle without a Helmet	History of Vomiting	Hematoma
0	Female	Linear Skull Fracture Subdural Hematoma	Hospitalization ≥ 2 nights	+	−	+	−	+	−	−
0	Male	Concussion	Hospitalization ≥ 2 nights	−	+	+	−	−	+	+
2	Male	Subarachnoid hemorrhage	Hospitalization ≥ 2 nights	−	+	−	−	−	+	−
3	Male	Concussion	Hospitalization ≥ 2 nights	+	+	+	+	−	−	−
5	Female	Linear skull fracture Pneumocephalus Epidural hematoma	Hematoma evacuation	−	−	+	−	−	+	−
9	Female	Concussion	Hospitalization ≥ 2 nights	+	−	+	+	−	−	−
15	Male	Concussion	Hospitalization ≥ 2 nights	+	−	+	+	−	−	−
15	Male	Linear skull fracture Pneumocephalus Epidural hematoma	Hospitalization ≥ 2 nights	+	−	−	+	−	−	−

Abbreviations: CATCH, Canadian Assessment of Tomography for Childhood Head Injury; CDR, clinical decision rule; ciTBI, clinically important traumatic brain injury; CHALICE, Children’s Head Injury Algorithm for the Prediction of Important Clinical Events; MVC, motor vehicle crash; PECARN, Pediatric Emergency Care Applied Research Network.

**Table 5:** Association between the number of applicable predictors in simplified CDR and ciTBI.

	ciTBI (+)	ciTBI (−)
	(*n* = 39)	(*n* = 266)
Number of CDR’s predictors, *n* (%)		
0	8 (20.5)	135 (50.8)
1	13 (33.3)	102 (38.3)
≥2	18 (46.2)	29 (10.9)

Abbreviations: ciTBI, clinically important traumatic brain injury.

## Data Availability

The datasets used and/or analyzed during the current study are available from the corresponding author on reasonable request.

## Appendix A

**Table A1:** Variables of CATCH, CHALICE, and PECARN.

Variables	CATCH	CHALICE	PECARN	Summarized Similar Variables
**Mechanism**	MVC	MVC as occupant, pedestrian, or cyclist greater than 40 miles/h	MVC with patient ejection, death of another passenger, or rollover	MVC
	Fall from bicycle with no helmet		Pedestrian or bicyclist without helmet struck by motorized vehicle	Fall from bicycle with no helmet
	Fall from elevation ≥3 ft	Fall > 3 m	Fall > 0.9 m (<2 years)/>1.5 m (>2 years)	Fall > 0.9 m
	Fall from ≥5 stairs			Fall from ≥5 stairs
		High-speed injury from a projectile or an object	Head struck by high-impact object	Head struck by high-impact object
**History**		Suspicion of non-accidental injury		Suspicion of non-accidental injury
	History of worsening headache		Severe headache (>2 years)	Severe or worsening headache
		≥3 vomits after head injury	History of vomiting (>2 years)	History of vomiting
		Seizure in patients with no history of epilepsy		Seizure
		Amnesia > 5 min	Not acting normally per parent (<2 years)	Amnesia
		LOC > 5 min	LOC > 5 s (<2 years)/Any or suspected LOC (>2 years)	LOC
**Examination**	GCS < 15 at 2 h after injury	GCS < 14, or GCS < 15 if aged < 1 year	GCS < 15	GCS < 15
	Irritability on examination			Irritability on examination
	Suspected open or depressed skull fracture Any sign of basal skull fracture	Signs of basal skull fracture Suspicion of penetrating or depressed skull injury or tense fontanelle	Palpable or unclear skull fracture (<2 years) Clinical signs of basal skull fracture (>2 years)	Any signs of skull fracture
	Large, boggy hematoma of the scalp	Presence of bruise, swelling or laceration > 5 cm if aged < 1 year	Occipital, parietal, or temporal scalp hematoma (<2 years)	Hematoma
		Abnormal drowsiness	Altered mental status	Altered mental status
		Positive focal neurology		Positive focal neurology

CATCH, Canadian Assessment of Tomography for Childhood Head Injury; CHALICE, Children’s Head Injury Algorithm for the Prediction of Important Clinical Events; GCS, Glasgow Coma Scale; LOC, loss of consciousness; MVC, motor vehicle crash; PECARN, Pediatric Emergency Care Applied Research Network.
